# The long-term effect of acupuncture for patients with chronic tension-type headache: study protocol for a randomized controlled trial

**DOI:** 10.1186/s13063-017-2188-9

**Published:** 2017-10-03

**Authors:** Lingyun Lu, Hui Zheng, Qianhua Zheng, Xinyu Hao, Siyuan Zhou, Shusen Zhang, Tao Wei, Tao Gao, Duoxi Duan, Ling Zhao, Ning Li, Ying Li

**Affiliations:** 1 Chengdu University of Traditional Chinese Medicine, 37 Shi’er Qiao Road, Jinniu District, Chengdu, Sichuan 610075 People’s Republic of China; 2 West China School of Medicine/West China Hospital of Sichuan University, 37 Guoxue Lane, Wuhou District, Chengdu, Sichuan 610041 People’s Republic of China

**Keywords:** Acupuncture, Chronic tension-type headache, Randomized controlled trial, Study protocol

## Abstract

**Background:**

The effectiveness of acupuncture for patients with chronic tension-type headache (CTTH) is controversial. In this article, we report the protocol for a randomized controlled trial aiming to evaluate the long-term effect of acupuncture for CTTH, in comparison with superficial acupuncture.

**Design:**

A two-armed, parallel-design, patient-assessor blind, randomized controlled trial is underway in China. A total of 218 participants with CTTH will be randomly assigned to the treatment group or the control group in a 1:1 ratio. Participants in the treatment group and control group will receive acupuncture or superficial acupuncture treatments in a fixed prescription of acupoints respectively, for a total of 20 sessions over 8 weeks. The posttreatment follow-up period will be 24 weeks. The primary outcome will be the proportion of responders assessed at week 16 after randomization. The secondary outcomes will include the number of headache days, the mean intensity of headache, the reduction of medication intake, results from the 36-item short form health survey, the Hamilton Depression Scale and the Hamilton Anxiety Scale, the number of participants with adverse events, the expectation value of acupuncture treatment, and the intensity of *deqi* sensation. The first five secondary outcomes will be assessed or calculated at baseline, and at 4, 8, 12, 16, 20, 24, 28, and 32 weeks after randomization. Moreover, the expectation value will be collected at baseline and at week 8 after randomization, the intensity of *deqi* sensation will be assessed at 5 minutes after each treatment and adverse events will be summarized at the end of the follow-up period.

**Discussion:**

Results from this trial will provide evidence for the long-term effect of acupuncture for CTTH with a long follow-up period.

**Trial registration:**

ClinicalTrial.gov NCT03133884. Registered on 25 April 2017.

**Electronic supplementary material:**

The online version of this article (doi:10.1186/s13063-017-2188-9) contains supplementary material, which is available to authorized users.

## Background

Chronic tension-type headache (CTTH) is a disorder evolving from frequent episodic tension-type headache (TTH), typically bilateral, pressing or tightening in quality, mild to moderate in intensity, lasting hours to days, or unremitting [1]. In April 2016, the World Health Organization reported that chronic headache, occurring on 15 or more days every month affected 1.7–4% of the world’s adult population, among which CTTH, as the most common chronic primary headache disorder, affected 1–3% of adults [2].

According to the guidelines developed by the European Federation of Neurological Societies, prophylactic treatment should be considered in patients with CTTH, and the recommended prophylactic drugs include amitriptyline, mirtazapine, venlafaxine, and muscle relaxants [3]. However, some unpleasant side effects of the recommended drugs have been reported, including sexual dysfunction, body weight changes, gastrointestinal discomfort, unusual bleeding, and bruising [4–6]. In addition to the serious adverse effects associated with the use of muscle relaxants, its evidence for application to CTTH patients is limited [7]. More importantly, issues such as medication overuse and how to prevent CTTH sufferers from developing medication overuse headache require greater attention.

Acupuncture, one of the most important components in traditional Chinese medicine (TCM), is widely used to relieve tension-type headache [3, 8]. Several trials of good quality have drawn the conclusion that acupuncture was superior to sham acupuncture in TTH treatment [9, 10]; some studies, however, have indicated no significant differences [11–13]. According to the National Institute for Health and Clinical Excellence clinical guidelines [8] and Cochrane Library systematic reviews [14, 15], acupuncture is recommended for CTTH management. Nevertheless, its scientific basis is still relatively limited owing to a lack of adequate statistical power, insufficient reports, or relevant methodological shortcomings in previous studies [16, 17].

CTTH is a chronic disorder characterized by persistent or recurrent attacks of headaches, thus the long-term effect of acupuncture is of particular importance because of its instant effect in treatments. Therefore, we have designed a randomized controlled trial (RCT) with a 36-week observation period to investigate and evaluate the long-term effect of acupuncture for CTTH.

## Method and design

### Study design

This study is a parallel-design, patient-assessor blind RCT comparing an acupuncture treatment group with a superficial acupuncture control group. Two hundred and eighteen patients with CTTH will be recruited through but not limited to: reviewing and screening of outpatients in the Teaching Hospital of Chengdu University of Traditional Chinese Medicine, university media releases, community advertisement including distributing leaflets via regular community health counselling, media campaigns, and network recruitment.

**Fig. 1:**
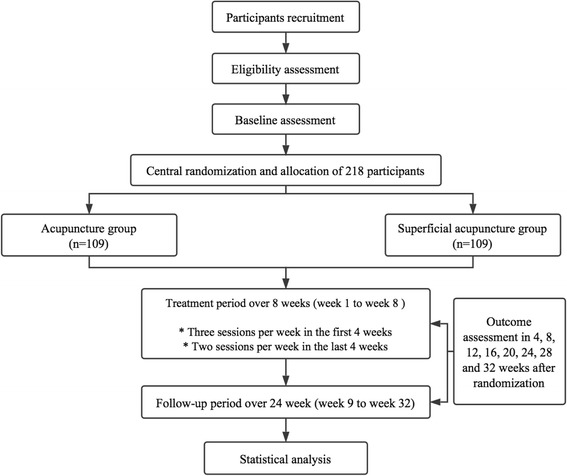
Flow chart of this trial

**Fig. 2:**
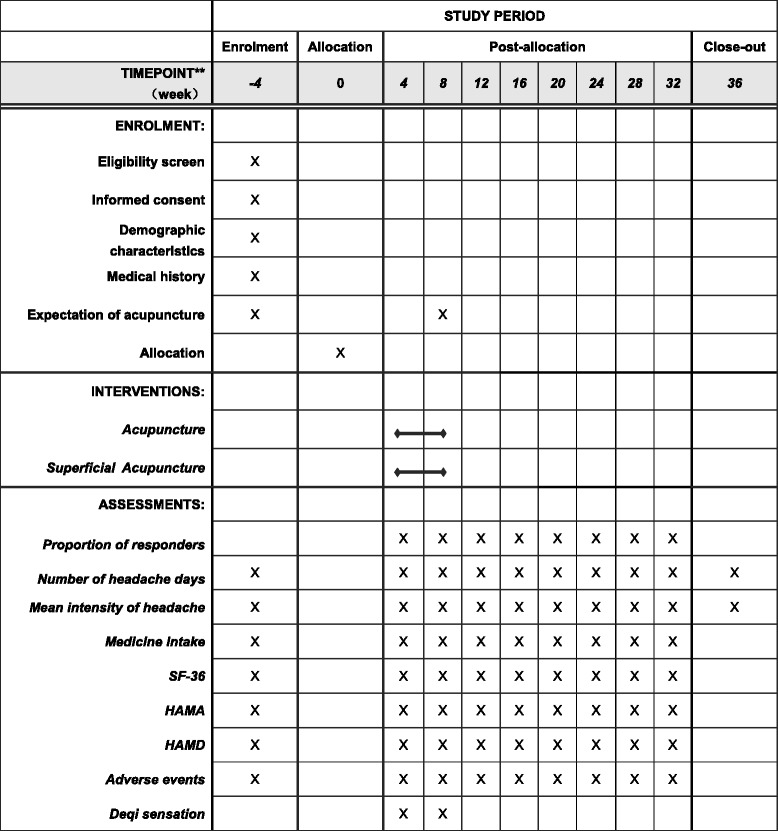
SPIRIT figure of enrolment, interventions, and assessments using the 36-item short form health survey (SF-36), Hamilton Depression Scale (HAMD) and Hamilton Anxiety Scale (HAMA). *Deqi* sensation will be assessed at 5 min after each treatment

Eligible participants will be randomly assigned to the acupuncture treatment group or the superficial acupuncture control group with a 1:1 ratio. The total observation period will be 36 weeks, including a 4-week baseline period, an 8-week treatment period and a 24-week follow-up period. They will receive 20 sessions of acupuncture or superficial acupuncture treatment over 8 weeks (three sessions per week in the first 4 weeks and two sessions per week in the following 4 weeks). During the baseline, treatment and follow-up periods, participants will not be allowed to take any prophylactic medications (e.g., amitriptyline, clomipramine, and mirtazapine) for CTTH, but will be permitted to use necessary analgesics (e.g., ibuprofen, paracetamol and ibuprofen) when headache is unbearable The type, dose and time of administration will be recorded in headache diaries. Assessments will be conducted at baseline and at 4, 8, 12, 16, 20, 24, 28, 32, and 36 weeks after randomization. Figures 1 and 2 illustrate the time schedule of enrolment, interventions, assessments, and visits of participants. The reporting of this trial is conducted according to the Standard Protocol Items: Recommendations for Intervention Trials (SPIRIT) guidelines (Additional file 1).

The Consolidated Standards of Reporting Trials [18] and the Standards for Reporting Interventions in Clinical Trials of Acupuncture [19] have been used as frameworks of methodology for designing this protocol.

### Participants

#### Study population and sample size

We will recruit CTTH sufferers who meet the diagnostic criteria in the International Classification of Headache Disorders, 3rd Edition (ICHD-3) formulated by the International Headache Society [1]. According to a previous study [11], the proportion of responders (at least 50% reduction in days with headache) was 46% after acupuncture treatment versus 35% after superficial acupuncture (odds ratio, 1.32). In this study, we expect an odds ratio of 1.35 when acupuncture is compared with superficial acupuncture. Assuming a significance level of 0.05, a study power of 0.8, and a correlation of 0.5 between independent variables (including study groups, assessment intervals, and the number of headache days at baseline), 199 participants are required to reject the hypothesis that acupuncture is equivalent to superficial acupuncture. Taking a 10% attrition bias into consideration, we will recruit 218 participants in total, with 109 in each group.

#### Inclusion criteria

Eligible participants should conform to ICHD-3 diagnostic criteria of CTTH and those who meet the following inclusion criteria will be included: (1) aged 18–65 years; (2) having TTH for more than 1 year; (3) suffering from TTH for at least 15 days per month on average during the previous 3 month; (4) able to complete the headache diary; (5) agree to participate in this study and provide written informed consent.

#### Exclusion criteria

Participants meeting any of the following criteria will be excluded: (1) headache is caused by other medical disorders (e.g., subarachnoid hemorrhage, cerebral hemorrhage, cerebral embolism, cerebral thrombosis, vascular malformation, arthritis, hypertension, or arteriosclerosis); (2) have taken prophylactic headache medication during the previous 3 months; (3) suffering from neurological diseases, mental disorders, immunodeficiency, bleeding disorders, or allergies; (4) having any serious disease of the heart, liver, kidney, or other organs; (5) those who are pregnant or lactating, or plan to be pregnant in the next 36 weeks; (6) unwilling to take part in the study or with low compliance, defined as the people who just plan to receive up to ten sessions of a total of 20 treatment sessions; (7) addicted to smoking, alcohol, or drugs; (8) involved in other clinical studies at the same time.

#### Dropout criteria

Participants will be removed if they are unwilling to continue this study, or fail to continue for at least 12 sessions of treatment.

### Randomization, allocation concealment and blinding

The Brightech Clinical Information Management System (CIMS) will be used for central randomization, which is performed by Brightech Clinical Medicine Research Co. Ltd. in Chengdu, China. Only authorized research assistants can obtain random numbers and group assignment information by logging into the CIMS website, which can ensure the randomization will not be influenced by researchers and patients. The randomization information will be concealed in the server until the end of the trial.

It is not possible to blind acupuncturists in this trial, therefore, they will not take part in the assessment procedure. Participants in different groups will receive treatment in different therapeutic rooms and be blinded to their treatment allocations. After all the treatment sessions, they will be asked to guess what kind of acupuncture treatment they have received (acupuncture or superficial acupuncture). Outcome assessors and statisticians will be blinded to the treatment allocation.

### Interventions

Combining Chinese acupuncture experts’ opinions with the results from data mining analysis of acupoints, a standardized acupoints prescription consisting of *Fengchi* (GB20), *Baihui* (GV20), *Taiyang* (EX-HN5), *Hegu* (LI4), and *Taichong* (LR3) will be applied for CTTH treatment in this trial (Table 1). Participants in both groups will receive 20 sessions of acupuncture over 8 weeks (three sessions per week in the first 4 weeks and two sessions per week in the last 4 weeks). Acupuncturists who have at least 5 years of professional training will receive training classes before this trial. Contents of the training include acupoint locating, needle manipulation skills, and communication skills. The acupuncturists can participate in the trial if they pass the trial training examination.

**Table 1:** Details of acupuncture and superficial acupuncture treatment

Group	Acupoints	Manipulation
Acupuncture	*Fengchi* (GB20)	0.5–0.8 *cun* (12.5–20 mm) oblique insertion towards the tip of the nose.
	*Baihui* (GV20)	0.5–0.8 *cun* (12.5–20 mm) subcutaneous insertion.
	*Taiyang* (EX-HN5)	0.3–0.5 *cun* (7.5–12.5 mm) perpendicular insertion.
	*Hegu* (LI4)	0.5–0.8 *cun* (12.5–20 mm) perpendicular insertion.
	*Taichong* (LR3)	0.5–0.8 *cun* (12.5–20 mm) perpendicular insertion.
		Retaining needles for 30 min. Lifting, thrusting, twisting and/or rotating the needles twice every 10 min with intermittent stimulation for maintaining the *deqi* sensation.
Superficial acupuncture	*Fengchi* (GB20)	Controlling depth within 2 mm by inserting needles with limited tubes, and retaining needles for 30 min without any manipulation to avoid *deqi* sensation as far as possible.
	*Baihui* (GV20)	
	*Taiyang* (EX-HN5)	
	*Hegu* (LI4)	
	*Taichong* (LR3)	

#### Acupuncture treatment group

All acupoints will be punctured by filiform needles (sterile, disposable needles provided by Suzhou Hwato Medical Instruments Co. Ltd., Suzhou, China) when patients are in a comfortable sitting position. The needles of 25 to 45 mm in length and 0.25 mm in diameter will be inserted into the acupoints and the depths will be adjusted to the standard permissible layers of each acupoint. Then an even reinforcing-reducing technique, which means lifting, thrusting, twisting, and rotating the needles moderately, will be performed on the needles until achieving the *deqi* sensation. The needles will be retained for 30 minutes in each session and manipulated every 10 minutes with intermittent stimulation for maintaining the *deqi* sensation. The manipulation of each acupoint will last for 10 seconds.

#### Superficial acupuncture control group

Participants in the control group will receive superficial acupuncture using filiform needles on the same acupoints as the treatment group. The depth of penetration into the skin will be controlled to within 2 mm by limited tubes, and the needles will be retained for 30 minutes without any manipulation to avoid the *deqi* sensation as much as possible.

### Outcome measurements

The following outcomes will be assessed by independent assessors blinded to the allocation.

#### Primary outcome

The primary outcome measurement is the proportion of responders at week 16. A responder is defined as a participant with a decrease of at least 50% less headache days during 4 weeks. A participant with a reduction of less than 50% headache days, including anyone with missing data, is defined as a nonresponder. This outcome measurement will be assessed at week 16 after randomization.

#### Secondary outcome

The secondary outcome measurements are (1) number of headache days per 4 weeks, (2) the mean intensity of headache recorded by visual analogue scale score and grade of headache intensity per 4 weeks, (3) the reduction of medication intake per 4 weeks, (4) the 36-item short form health survey (SF-36) [20], (5) Hamilton Depression Scale (HAMD) [21] and Hamilton Anxiety Scale (HAMA) [22] results, (6) number of participants with adverse events (AEs) and serious adverse events (SAEs), (7) expectation values of acupuncture treatment, (8) the intensity of the *deqi* sensation.

All participants will be asked to complete a daily headache dairy and return them to assessors by emails, short messages or telephone calls throughout the whole 36-week observation period. The first three secondary outcomes will be extracted from the headache diaries. And the three outcomes followed will be assessed at each visiting time point. All of the above outcome measurements will be assessed at baseline, and at 4, 8, 12, 16, 20, 24, 28, and 32 weeks after randomization. Moreover, the expectation value of acupuncture treatment will be collected at baseline and week 8 after randomization, and the intensity of the *deqi* sensation will be assessed at 5 minutes after each treatment using the Massachusetts General Hospital Acupuncture Sensation Scale (MASS).

Details of AEs and SAEs will be documented during the 36-week period, such as occurrence time, duration, coping methods, and the correlation with treatment. AEs include bleeding, hematoma, fainting, severe pain, local infection, and so on, while SAEs are defined as life-threatening events, leading to significant or persisted disability, resulting in or prolonging hospitalization.

Researchers responsible for recruiting participants and assessors responsible for collecting data must receive training to ensure all of them have the same standards and understand the purpose, contents, and quality control of the trial. Detailed time points of outcome assessments are provided in Fig. 2.

### Data collecting and monitoring

Data will be recorded on the paper version of case report forms (CRFs) by designated outcome assessors, and double entered in the electronic CRFs, which is established and monitored by the Evidence-based Medicine Center of Chengdu University of Traditional Chinese Medicine. Monitors will audit the data every 3 months. Acupuncturists and statisticians will have no access to these data during the evaluating process.

### Statistical analysis

Primary analysis will be based on intention-to-treat population, including participants with at least one assessment of the primary outcome and one acupuncture session. Per protocol (PP) analysis will be performed to test the robustness of the primary analysis. PP population will include participants receiving at least 12 sessions of acupuncture and be assessed at week 8 and 16.

The primary outcome will be analyzed with a random intercept logistic regression model, taking treatment allocation as a random factor and taking sex, age, and duration of headache as fixed factors. The secondary outcomes (headache days, headache intensity, SF-36, HAMA, HAMD results) will be analyzed using a generalized least squares regression model, with the same covariates as the primary outcome. For the rest of outcomes, we will make pairwise comparisons using a general linear model adjusted for baseline value, age, sex, and disease course. Missing values of the primary outcome will be imputed by the last-observation-carried-forward method. We will run several sensitivity analysis to ensure the robustness of the imputation: first, lost-to-follow-up participants in the acupuncture group will be recognized as nonresponders, whereas those in the superficial acupuncture group will be recognized as responders; second, lost-to-follow-up participants will be counted as responders in the acupuncture group but nonresponders in the superficial group; Third, all lost-to-follow-up participants will be treated as nonresponders. Missing values in the secondary outcomes will be imputed by using the multiple imputation method (*n* = 5).

All confidence intervals will be two-sided 95% intervals comparing acupuncture to superficial acupuncture and all statistical hypotheses will be two-sided tests. Analyses will be performed using the R and Stata programs.

## Discussion

Previous RCTs have been conducted to explore the effect of acupuncture for TTH compared with no treatment [11, 23], sham interventions (non-skin-penetrating techniques or needling non-acupoints) [9, 11–13, 16], or other treatment (relaxation, massage, or physiotherapy) [17, 26–28], but the conclusions remain controversial. In a recent Cochrane systematic review [15], seven of the 12 included trials recruited both episodic and chronic TTH patients [9, 11, 13, 23–26, 28], which made it relatively difficult to evaluate the effect of acupuncture for CTTH separately and precisely. Three of the 12 trials [16, 17, 27] included participants with CTTH only, but their small sample sizes, various types of acupuncture interventions, non-uniformed comparisons, and different observation periods may lead to an uncertain evaluation of acupuncture effect. Therefore, we designed a two-armed, parallel-design, patient-assessor blind, RCT to explore and evaluate the long-term effect of acupuncture for CTTH treatment. In this trial, we will compare acupuncture with superficial acupuncture to clarify the long-term therapeutic effect of acupuncture for CTTH.

In this trial, participants in different groups will receive treatments in different rooms to block the communication between the two groups and reduce patients’ doubts about different interventions. Moreover, all of the acupuncture manipulations will be performed on the fixed main acupoints by two trained acupuncturists who have passed the special training examination, in order to reduce the influence of participants’ psychological factors on clinical effects. It is reported that negative mood state has a close relationship with TTH [29, 30] Therefore, psychological assessments of participants based on HAMA and HAMD are planned. Furthermore, we will pay special attention to the recording of the *deqi* sensation, which may enhance the acupuncture effect as described in TCM classical works and traditional acupuncture theory [31].

A placebo control plays a crucial part in evaluating the effectiveness of a treatment, which can help separate the specific and nonspecific effects and reduce bias by enabling blinding. Therefore, being inert and indistinguishable from the true intervention are the main characteristics of a perfect placebo or a sham intervention. However, to devise or find adequate placebos seems too difficult for physical interventions, such as acupuncture. Currently, superficial acupuncture is considered as a type of sham acupuncture, because the needle depth does not reach acupoints’ anatomical layers. However, there is increasing evidence that sham acupuncture might be associated with larger placebo effects than pharmacological placebos [32–34], especially for some subjective outcomes and the treatment of pain [35]. A positive expectation of an intervention is an important part of the placebo effect [36, 37], which is also supported by many previous trials [38–41]. In addition, a good doctor-patient relationship also contributes to patients’ expectations, which may increase placebo effects after repeated treatments and harmonious communications [42]. It may be the reason why no significant differences of effects between acupuncture and sham acupuncture were reported by several trials [11–13, 24]. Hence, we will make a comparison of participants’ expectations between two groups to eliminate the impact on our results.

A recent RCT reported that the acupuncture effect can be maintained for 20 weeks after 20 sessions of treatment over 4 weeks, which could be considered as powerful evidence for the existence of a sustained effect produced by acupuncture [43]. In addition, a systematic review indicated that the placebo effect of acupuncture showed a unique pattern according to time, which increased from baseline to 12 weeks and decreased after 12 weeks [44]. Considering the necessity of a relatively long observation period, we will conduct the trial over a period of 36 weeks and make assessments every 4 weeks, which aims to not only evaluate the long-term effect of acupuncture for CTTH, but also distinguish curative effects from placebo effects of acupuncture. However, the long observation period has its limitations. It may increase the rate of patient dropout and we will not know if the patients receive other treatments in the follow-up period. To minimize the potential bias, we formulate the plan to reduce dropout by establishing good communication relationships with participants and maintaining close contacts via telephone calls, text messages and emails during the follow-up period. Moreover, characteristics of the dropouts will be extracted and analyzed, which can help judge whether the results are affected by the bias.

In conclusion, the results of this trial are expected to not only provide clinical evidence of the effectiveness of acupuncture as a painkiller, but also evaluate the duration and intensity of the acupuncture effect for CTTH.

### Trial status

The trial project commenced in early 2017. Recruitment of participants is currently underway.

## Additional file

Additional file 1: SPIRIT 2013 checklist: recommended items to address in a clinical trial protocol and related documents*. (DOC 124 kb)

## Abbreviations

AEs: adverse events
CRFs: Case report forms
CTTH: Chronic tension-type headache
HAMA: Hamilton Anxiety Scale
HAMD: Hamilton Depression Scale
ICHD-3: International Classification of Headache Disorders, 3rd Edition
RCT: randomized controlled trial
SF-36: 36-item short form health survey
SAEs: serious adverse events
SPIRIT: Standard Protocol Items: Recommendations for Intervention Trials
TCM: Traditional Chinese medicine
TTH: Tension-type headache
